# Supplementary material for: A Live Attenuated COVID-19 Candidate Vaccine for Children: Protection against SARS-CoV-2 Challenge in Hamsters
Source: Vaccines (Basel). 2023 Jan 24;11(2):255. doi: 10.3390/vaccines11020255 (PMC9965573; doi:10.3390/vaccines11020255)
Supplement: Supplementary file 1 [file vaccines-11-00255-s001.zip › S1 Histopathological observations.pdf]

Supplementary data S1. Histopathological observations

Summary Report of Histopathology in Hamsters after administration of COVID-19 vaccine via intranasal and intramuscular route

Organs samples collected on day 6:

Six to seven weeks old Hamsters were grouped according to the planned route of vaccine administration and vaccine dose as shown below.

Groups	Dose in hamsters (Route)	Route of administration	No of animals
Group 1	6.0 Log PFU	IN	3
Group 2	6.0 Log PFU	IM	5
Group 3	5.0 Log PFU	IM	5
Group 4	Placebo	IM	3

On day 6 post administration of COVID-19 vaccine via intramuscular / intranasal route animals were sacrificed and six organs per animal (Lungs, Trachea, Spleen, Kidney, Heart and Brain) were collected for histopathology.

Result:

1. Sparse mononuclear cell infiltrate was observed in Trachea in all animals irrespective of the treatment and was similar to Placebo group. Polymorphs were also observed in lungs, heart and kidney in all samples.
2. In animals administered with 6.0 Log_{10} PFU via intramuscular or intranasal route, tissue morphology was observed unremarkable and was similar to that of placebo group.

Conclusion:

No remarkable difference was observed attributable to any of the treatment groups.

Supplementary data S1. Histopathological observations

Histopathological findings on day 6 post vaccine administration:

Sr. no.	Groups ►	**Group 1 (Intranasal)** (Dose = 6.0 Log_{10} PFU)			**Group 2 (Intramuscular)** (Dose = 6.0 Log_{10} PFU)				
	**Animal codes►**	**RH**	**RT**	**GH**	**RH**	**RT**	**GH (Co-housed)**	**GT (Co-housed)**	**W**
**01.**	**Lungs:**								
	Size (cm)	1.2 X 1.0 X 0.5	1.5 X 1.0 X 0.5	1.3 X 1.0 X 0.5	1.3 X 1.0 X 0.8	1.0 X 1.8 X 0.6	1.2 X 1.0 X 0.6	1.0 x 0.8 x 0.6	1.0 x 0.8 x 0.5
	Architecture	Unremarkable	Unremarkable	Unremarkable	Unremarkable	Unremarkable	Unremarkable	Unremarkable	Unremarkable
	Alveoli	Unremarkable	Unremarkable	Unremarkable	Unremarkable	Unremarkable	Unremarkable	Unremarkable	Unremarkable
	Stroma	Moderate diffuse mixed inflammatory cell infiltrate along with macrophages<br><br>60–70 polymorphs / hpf.	Mild diffuse mixed inflammatory cell infiltrate along with macrophages.<br><br>50–60 polymorphs / hpf.	Moderate diffuse mixed inflammatory cell infiltrate along with macrophages.<br><br>>200 polymorphs / hpf.	Moderate diffuse mixed inflammatory cell infiltrate along with macrophages. 25–30 polymorphs / hpf.	Moderate diffuse mixed inflammatory cell infiltrate along with macrophages. 70–80 polymorphs / hpf.	Mild diffuse mixed inflammatory cell infiltrate along with macrophages.<br><br>40–50 polymorphs / hpf.	Moderate diffuse mixed inflammatory cell infiltrate along with macrophages.<br><br>130–150 polymorphs / hpf	Mild diffuse mixed inflammatory cell infiltrate along with macrophages.<br><br>50–60 polymorphs / hpf.
	Micro-abscesses	Not seen	Not seen	Not seen	Not seen	Not seen	Not seen	Not seen	Not seen
	Alveolar exudate/cells	Not seen	Not seen	Not seen	Not seen	Not seen	Not seen	Not seen	Not seen
	Stromal infiltrate	Seen	Seen	Seen	Seen	Seen	Seen	Seen	Seen
	Other cells (infiltrate)	No granulomas seen	No granulomas seen	No granulomas seen	No granulomas seen	No granulomas seen	No granulomas seen	No granulomas seen	No granulomas seen
**02.**	**Trachea:**								
	Length (cm)	0.6	1.0	1.2	0.6	0.6	0.5	0.6	0.8
	Mucosa	Sparse mononuclear cell infiltrate	Sparse mononuclear cell infiltrate	Sparse mononuclear cell infiltrate	Sparse mononuclear cell infiltrate	Sparse mononuclear cell infiltrate	Sparse mononuclear cell infiltrate	Sparse mononuclear cell infiltrate	Sparse mononuclear cell infiltrate
	Submucosa	Unremarkable	Unremarkable	Unremarkable	Unremarkable	Unremarkable	Unremarkable	Unremarkable	Unremarkable
	Trachealis muscle	Unremarkable	Unremarkable	Unremarkable	Unremarkable	Unremarkable	Unremarkable	Unremarkable	Unremarkable

Supplementary data S1. Histopathological observations

Sr. no.	Groups ►	Group 1 (Intranasal) (Dose = 6.0 Log_{10} PFU)			Group 2 (Intramuscular) (Dose = 6.0 Log_{10} PFU)				
	**Animal codes►**	**RH**	**RT**	**GH**	**RH**	**RT**	**GH (Co-housed)**	**GT (Co-housed)**	**W**
	Hyaline cartilage	Unremarkable	Unremarkable	Unremarkable	Unremarkable	Unremarkable	Unremarkable	Unremarkable	Unremarkable
	Adventitia	Unremarkable	Unremarkable	Unremarkable	Unremarkable	Unremarkable	Unremarkable	Unremarkable	Unremarkable
**03.**	**Brain:**								
	Size (cm)	1.0 X 0.8 X 0.6	1.5 X 1.0 X 0.8	1.8 x 1.2 X 1.0	1.3 X 1 X 0.8	1.3 X 1.0 X 1.0	0.9 X 0.7 X 0.6	1.2 X 1.0 X 0.8	1.2 X 1.0 X 0.8
	Architecture	Unremarkable	Unremarkable	Unremarkable	Unremarkable	Unremarkable	Unremarkable	Unremarkable	Unremarkable
	Cortex/Greymatter	Unremarkable	Unremarkable	Unremarkable	Unremarkable	Unremarkable	Unremarkable	Unremarkable	Unremarkable
	Subcortical white matter	Unremarkable	Unremarkable	Unremarkable	Unremarkable	Unremarkable	Unremarkable	Unremarkable	Unremarkable
	Blood vessels	Unremarkable	Unremarkable	Unremarkable	Unremarkable	Unremarkable	Unremarkable	Unremarkable	Unremarkable
	Granulomas	Not seen	Not seen	Not seen	Not seen	Not seen	Not seen	Not seen	Not seen
**04.**	**Heart:**								
	Size (cm)	0.7 X 0.6 X 0.5	1.0 X 0.8 X 0.6	0.9 X 0.7 X 0.6	1.0 X 0.8 X 0.6	0.9 X 0.8 X 0.7	0.6 X 0.5 X 0.5	0.9 X 0.7 X 0.5	0.9 X 0.7 X 0.5
	Epicardium	Unremarkable	Unremarkable	Unremarkable	Unremarkable	Unremarkable	Unremarkable	Unremarkable	Unremarkable
	Myocardium	Sparse diffuse mononuclear cell infiltrate	Sparse diffuse mononuclear cell infiltrate	Sparse diffuse mononuclear cell infiltrate	Sparse diffuse mononuclear cell infiltrate	Sparse diffuse mononuclear cell infiltrate	Sparse diffuse mononuclear cell infiltrate	Sparse diffuse mononuclear cell infiltrate	Sparse diffuse mononuclear cell infiltrate
	Endocardium	Unremarkable	Unremarkable	Unremarkable	Unremarkable	Unremarkable	Unremarkable	Unremarkable	Unremarkable
	Chambers	Unremarkable	Unremarkable	Unremarkable	Unremarkable	Unremarkable	Unremarkable	Unremarkable	Unremarkable
	Vessels	Unremarkable	Unremarkable	Unremarkable	Unremarkable	Unremarkable	Unremarkable	Unremarkable	Unremarkable
05.	**Kidney:**								
	Size (cm)	1.5 X 1.0 X 0.8	1.3 X 1.0 X 0.8	1.5 X 1.0 X 0.8	1.5 X 1.0 X 0.8	1.5 X 1.2 X 1.0	1.3 X 1.0 X 0.8	1.5 X 1.0 X 0.8	1.4 X 1.0 X 0.8
	Capsule	Unremarkable	Unremarkable	Unremarkable	Unremarkable	Unremarkable	Unremarkable	Unremarkable	Unremarkable
	Architecture	Maintained	Maintained	Maintained	Maintained	Maintained	Maintained	Maintained	Maintained
	Glomeruli	No significant pathology	No significant pathology	No significant pathology	No significant pathology	No significant pathology	No significant pathology	No significant pathology	No significant pathology
	Tubules	Unremarkable	Unremarkable	Unremarkable	Unremarkable	Unremarkable	Unremarkable	Unremarkable	Unremarkable
	Vessels	No significant pathology	No significant pathology	No significant pathology	No significant pathology	No significant pathology	No significant pathology	No significant pathology	No significant pathology

Supplementary data S1. Histopathological observations

Sr. no.	Groups ►	Group 1 (Intranasal) (Dose = 6.0 Log_{10} PFU)			Group 2 (Intramuscular) (Dose = 6.0 Log_{10} PFU)				
	**Animal codes►**	**RH**	**RT**	**GH**	**RH**	**RT**	**GH (Co-housed)**	**GT (Co-housed)**	**W**
	Interstitium infiltrate	Sparse mononuclear cell infiltrate	Sparse mononuclear cell infiltrate	Sparse mononuclear cell infiltrate	Sparse mononuclear cell infiltrate	Sparse mononuclear cell infiltrate	Sparse mononuclear cell infiltrate	Sparse mononuclear cell infiltrate	Sparse mononuclear cell infiltrate
06.	**Spleen:**								
	Size (cm)	0.8 X 0.5 X 0.2	1.0 X 0.5 X 0.2	1.0 X 0.5 X 0.2	1.3 X 1.0 X 0.8	1.0 X 0.8 X 0.2	1.0 X 0.5 X 0.2	1.0 X 0.5 X 0.2	0.6 X 0.5 X 0.2
	Capsule	Intact	Intact	Intact	Intact	Intact	Intact	Intact	Intact
	Architecture	Maintained	Maintained	Maintained	Maintained	Maintained	Maintained	Maintained	Maintained
	Red pulp	Prominent	Prominent	Prominent	Prominent	Prominent	Prominent	Prominent	Prominent
	White pulp	Lymphoid follicles with secondary germinal centres	Lymphoid follicles with secondary germinal centres	Lymphoid follicles with secondary germinal centres	Lymphoid follicles with secondary germinal centres	Lymphoid follicles with secondary germinal centres	Lymphoid follicles with secondary germinal centres	Lymphoid follicles with secondary germinal centres	Lymphoid follicles with secondary germinal centres
	Fibrosis tissue	Not seen	Not seen	Not seen	Not seen	Not seen	Not seen	Not seen	Not seen
	Granulomas	Not seen	Not seen	Not seen	Not seen	Not seen	Not seen	Not seen	Not seen
	Necrosis	Not seen	Not seen	Not seen	Not seen	Not seen	Not seen	Not seen	Not seen
	Atypical cells	Not seen	Not seen	Not seen	Not seen	Not seen	Not seen	Not seen	Not seen

Sr. no.	Groups ►	Group 3 (Intramuscular) (Dose = 5.0 Log_{10} PFU)					Group 4 (Placebo)		
	**Animal codes►**	**RH**	**RT**	**GH (Co-housed)**	**GT (Co-housed)**	**W**	**RH**	**RT**	**GH**
**01.**	**Lungs:**								
	Size (cm)	1.0 X 0.8 X 0.5	1.0 X 0.8 X 0.6	1.3 X 1.0 X 0.7	1.0 X 0.8 X 0.7	1.4 X 1.0 X 0.8	1.5 X 1.0 X 0.8	1.2 x 1.0 x 0.7	1.0 x 0.8 x 0.6
	Architecture	Unremarkable	Unremarkable	Unremarkable	Unremarkable	Unremarkable	Unremarkable	Unremarkable	Unremarkable
	Alveoli	Unremarkable	Unremarkable	Unremarkable	Unremarkable	Unremarkable	Unremarkable	Unremarkable	Unremarkable

Supplementary data S1. Histopathological observations

Sr. no.	Groups ►	Group 3 (Intramuscular) (Dose = 5.0 Log_{10} PFU)					Group 4 (Placebo)		
	Animal codes►	RH	RT	GH (Co-housed)	GT (Co-housed)	W	RH	RT	GH
	Stroma	Moderate diffuse mixed inflammatory cell infiltrate along with macrophages.<br><br>60–70 polymorphs / hpf.	Mild diffuse mixed inflammatory cell infiltrate along with macrophages.<br><br>30–35 polymorphs / hpf.	Moderate diffuse mixed inflammatory cell infiltrate along with macrophages.<br><br>60–70 polymorphs / hpf.	Mild diffuse mixed inflammatory cell infiltrate along with macrophages.<br><br>30–35 polymorphs / hpf.	Mild diffuse mixed inflammatory cell infiltrate along with macrophages.<br><br>60–70 polymorphs / hpf.	Moderate diffuse mixed inflammatory cell infiltrate along with macrophages.<br><br>60–70 polymorphs / hpf.	Moderate diffuse mixed inflammatory cell infiltrate along with macrophages.<br><br>40–50 polymorphs / hpf.	Mild diffuse mixed inflammatory cell infiltrate along with macrophages.<br><br>30–40 polymorphs / hpf.
	Micro-abscesses	Not seen	Not seen	Not seen	Not seen	Not seen	Not seen	Not seen	Not seen
	Alveolar exudate/cells	Not seen	Not seen	Not seen	Not seen	Not seen	Not seen	Not seen	Not seen
	Stromal infiltrate	Seen	Seen	Seen	Seen	Seen	Seen	Seen	Seen
	Other cells (infiltrate)	No granulomas seen	No granulomas seen	No granulomas seen	No granulomas seen	No granulomas seen	No granulomas seen	No granulomas seen	No granulomas seen
**02.**	**Trachea:**								
	Length (cm)	0.5	0.6	0.8	0.6	0.8	1.0	0.9	0.6
	Mucosa	Sparse mononuclear cell infiltrate	Sparse mononuclear cell infiltrate	Sparse mononuclear cell infiltrate	Sparse mononuclear cell infiltrate	Sparse mononuclear cell infiltrate	Sparse mononuclear cell infiltrate	Sparse mononuclear cell infiltrate	Sparse mononuclear cell infiltrate
	Submucosa	Unremarkable	Unremarkable	Unremarkable	Unremarkable	Unremarkable	Unremarkable	Unremarkable	Unremarkable
	Trachealis muscle	Unremarkable	Unremarkable	Unremarkable	Unremarkable	Unremarkable	Unremarkable	Unremarkable	Unremarkable
	Hyaline cartilage	Unremarkable	Unremarkable	Unremarkable	Unremarkable	Unremarkable	Unremarkable	Unremarkable	Unremarkable
	Adventitia	Unremarkable	Unremarkable	Unremarkable	Unremarkable	Unremarkable	Unremarkable	Unremarkable	Unremarkable
03.	**Brain:**								
	Size (cm)	1.2 X 1.0 X 0.8	1.4 X 1.2 X 1.0	1.2 x 1.0 X 0.8	1.3 X 1.0 X 0.8	1.2 X 1.0 X 0.8	1.5 X 1.0 X 0.7	1.0 X 0.9 X0.7	1.2 X 1.0 X0.8
	Architecture	Unremarkable	Unremarkable	Unremarkable	Unremarkable	Unremarkable	Unremarkable	Unremarkable	Unremarkable

Supplementary data S1. Histopathological observations

Sr. no.	Groups ►	Group 3 (Intramuscular) (Dose = 5.0 Log_{10} PFU)					Group 4 (Placebo)		
	Animal codes►	RH	RT	GH (Co-housed)	GT (Co-housed)	W	RH	RT	GH
	Cortex/Greymatter	Unremarkable	Unremarkable	Unremarkable	Unremarkable	Unremarkable	Unremarkable	Unremarkable	Unremarkable
	Subcortical white matter	Unremarkable	Unremarkable	Unremarkable	Unremarkable	Unremarkable	Unremarkable	Unremarkable	Unremarkable
	Blood vessels	Unremarkable	Unremarkable	Unremarkable	Unremarkable	Unremarkable	Unremarkable	Unremarkable	Unremarkable
	Granulomas	Not seen	Not seen	Not seen	Not seen	Not seen	Not seen	Not seen	Not seen
04.	**Heart:**								
	Size (cm)	0.8 X 0.7 X 0.6	0.8 X 0.6 X 0.5	1.0 X 0.8 X 0.6	0.8 X 0.6 X 0.5	1.0 X 0.8 X 0.6	1.0 X 0.8 X 0.7	0.6 X 0.5 X 0.4	1.0 X 0.8 X 0.6
	Epicardium	Unremarkable	Unremarkable	Unremarkable	Unremarkable	Unremarkable	Unremarkable	Unremarkable	Unremarkable
	Myocardium	Sparse diffuse mononuclear cell infiltrate	Sparse diffuse mononuclear cell infiltrate	Sparse diffuse mononuclear cell infiltrate	Sparse diffuse mononuclear cell infiltrate	Sparse diffuse mononuclear cell infiltrate	Sparse diffuse mononuclear cell infiltrate	Sparse diffuse mononuclear cell infiltrate	Sparse diffuse mononuclear cell infiltrate
	Endocardium	Unremarkable	Unremarkable	Unremarkable	Unremarkable	Unremarkable	Unremarkable	Unremarkable	Unremarkable
	Chambers	Unremarkable	Unremarkable	Unremarkable	Unremarkable	Unremarkable	Unremarkable	Unremarkable	Unremarkable
	Vessels	Unremarkable	Unremarkable	Unremarkable	Unremarkable	Unremarkable	Unremarkable	Unremarkable	Unremarkable
05.	**Kidney:**								
	Size (cm)	1.0 X 1.0 X 1.0	1.5 X 1.0 X 0.8	1.5 X 1.0 X 0.8	1.5 X 1.0 X 0.8	1.5 X 1.0 X 0.7	1.4 X 1.0 X 0.8	1.2 X 1.0 X0.8	1.5 X 1.2X 1.0
	Capsule	Unremarkable	Unremarkable	Unremarkable	Unremarkable	Unremarkable	Unremarkable	Unremarkable	Unremarkable
	Architecture	Maintained	Maintained	Maintained	Maintained	Maintained	Maintained	Maintained	Maintained
	Glomeruli	No significant pathology	No significant pathology	No significant pathology	No significant pathology	No significant pathology	No significant pathology	No significant pathology	No significant pathology
	Tubules	Unremarkable	Unremarkable	Unremarkable	Unremarkable	Unremarkable	Unremarkable	Unremarkable	Unremarkable
	Vessels	No significant pathology	No significant pathology	No significant pathology	No significant pathology	No significant pathology	No significant pathology	No significant pathology	No significant pathology
	Interstitium infiltrate	Sparse mononuclear cell infiltrate	Sparse mononuclear cell infiltrate	Sparse mononuclear cell infiltrate	Sparse mononuclear cell infiltrate	Sparse mononuclear cell infiltrate	Sparse mononuclear cell infiltrate	Sparse mononuclear cell infiltrate	Sparse mononuclear cell infiltrate
06.	**Spleen:**								
	Size (cm)	0.6 X 0.5 X 0.2	0.6 X 0.5 X 0.2	1.0 X 0.5 X 0.2	0.7 X 0.5 X 0.2	0.9 X 0.5 X 0.2	0.8 X 0.5 X 0.2	0.8 X 0.5 X 0.2	0.9 X 0.5 X 0.2

Supplementary data S1. Histopathological observations

Sr. no.	Groups ►	Group 3 (Intramuscular) (Dose = 5.0 Log_{10} PFU)					Group 4 (Placebo)		
	**Animal codes►**	**RH**	**RT**	**GH (Co-housed)**	**GT (Co-housed)**	**W**	**RH**	**RT**	**GH**
	Capsule	Intact	Intact	Intact	Intact	Intact	Intact	Intact	Intact
	Architecture	Maintained	Maintained	Maintained	Maintained	Maintained	Maintained	Maintained	Maintained
	Red pulp	Prominent	Prominent	Prominent	Prominent	Prominent	Prominent	Prominent	Prominent
	White pulp	Lymphoid follicles with secondary germinal centres	Lymphoid follicles with secondary germinal centres	Lymphoid follicles with secondary germinal centres	Lymphoid follicles with secondary germinal centres	Lymphoid follicles with secondary germinal centres	Lymphoid follicles with secondary germinal centres	Lymphoid follicles with secondary germinal centres	Lymphoid follicles with secondary germinal centres
	Fibrosis tissue	Not seen	Not seen	Not seen	Not seen	Not seen	Not seen	Not seen	Not seen
	Granulomas	Not seen	Not seen	Not seen	Not seen	Not seen	Not seen	Not seen	Not seen
	Necrosis	Not seen	Not seen	Not seen	Not seen	Not seen	Not seen	Not seen	Not seen
	Atypical cells	Not seen	Not seen	Not seen	Not seen	Not seen	Not seen	Not seen	Not seen

Supplementary data S1. Histopathological observations

Organs samples collected on day 28:

Six to seven weeks old Hamsters were grouped according to the planned route of vaccine administration and vaccine dose as shown below.

Groups	Dose in hamsters	Route of administration	No of animals
Group 1	6.0 Log PFU	Intranasal (IN)	3
Group 2	6.0 Log PFU	Intramuscular (IM)	5
Group 3	5.0 Log PFU	Intramuscular (IM)	5
Group 4	Placebo	Intramuscular (IM)	3

On day 28 post administration of COVID-19 vaccine via intramuscular / intranasal route animals were sacrificed and six organs per animal (Lungs, Trachea, Spleen, Kidney, Heart and Brain) were collected for histopathology.

Result:

Sparse mononuclear cell infiltrate was observed in Trachea in all animals irrespective of the treatment and was similar to Placebo group. Polymorphs were also observed in lungs, heart and kidney in all samples.

In animals administered with 6.0 Log_{10} PFU via intranasal and intramuscular routes (group 1 and group 2), tissue morphology observed was unremarkable and found similar to that of placebo group.

Conclusion:

No remarkable histopathological abnormality was detected attributable to any of the treatment groups.

Supplementary data S1. Histopathological observations

Histopathological findings on day 28 post vaccine administration

Sr. no.	**Groups ►**	**Group 1 (Intranasal)** (Dose = 6.0 Log_{10} PFU)			**Group 2 (Intramuscular)**(Dose = 6.0 Log_{10} PFU)				
	**Animal codes►**	**RH**	**RT**	**GH**	**RH**	**RT**	**GH (Co-housed)**	**GT(Co-housed)**	**W**
**01.**	**Lungs:**								
	Size (cm)	1.4 X 1.0 X 0.8	1.3 X 1.0 X 0.8	1.0 X 0.8 X 0.7	1.2 X 0.8 X 0.7	1.3 X 1.0 X 0.8	1.2 X 1.0 X 0.6	1.4 x 1.0 x 1.0	1.3 x 1.0 x 0.8
	Architecture	Unremarkable	Unremarkable	Unremarkable	Unremarkable	Unremarkable	Unremarkable	Unremarkable	Unremarkable
	Alveoli	Unremarkable	Unremarkable	Unremarkable	Unremarkable	Unremarkable	Unremarkable	Unremarkable	Unremarkable
	Stroma	Moderate diffuse mixed inflammatory cell infiltrate along with macrophages<br><br>70–80 polymorphs / hpf.	Moderate diffuse mixed inflammatory cell infiltrate along with macrophages<br><br>70–80 polymorphs / hpf.	Moderate diffuse mixed inflammatory cell infiltrate along with macrophages.<br><br>120-140 polymorphs / hpf.	Moderate diffuse mixed inflammatory cell infiltrate along with macrophages. 90–100 polymorphs / hpf.	Moderate diffuse mixed inflammatory cell infiltrate along with macrophages. 100–120 polymorphs / hpf.	Mild diffuse mixed inflammatory cell infiltrate along with macrophages.<br><br>40–50 polymorphs / hpf.	Moderate diffuse mixed inflammatory cell infiltrate along with macrophages.<br><br>80–90 polymorphs / hpf	Moderate diffuse mixed inflammatory cell infiltrate along with macrophages.<br><br>130–150 polymorphs / hpf
	Micro-abscesses	Not seen	Not seen	Not seen	Not seen	Not seen	Not seen	Not seen	Not seen
	Alveolar exudate/cells	Not seen	Not seen	Not seen	Not seen	Not seen	Not seen	Not seen	Not seen
	Stromal infiltrate	Seen	Seen	Seen	Seen	Seen	Seen	Seen	Seen
	Other cells (infiltrate)	No granulomas seen	No granulomas seen	No granulomas seen	No granulomas seen	No granulomas seen	No granulomas seen	No granulomas seen	No granulomas seen
**02.**	**Trachea:**								
	Length (cm)	1.0	0.9	0.8	1.0	1.0	1.0	1.0	0.7
	Mucosa	Sparse mononuclear cell infiltrate	Sparse mononuclear cell infiltrate	Sparse mononuclear cell infiltrate	Sparse mononuclear cell infiltrate	Sparse mononuclear cell infiltrate	Sparse mononuclear cell infiltrate	Sparse mononuclear cell infiltrate	Sparse mononuclear cell infiltrate
	Submucosa	Unremarkable	Unremarkable	Unremarkable	Unremarkable	Unremarkable	Unremarkable	Unremarkable	Unremarkable
	Trachealis muscle	Unremarkable	Unremarkable	Unremarkable	Unremarkable	Unremarkable	Unremarkable	Unremarkable	Unremarkable

Supplementary data S1. Histopathological observations

Sr. no.	Groups ►	Group 1 (Intranasal) (Dose = 6.0 Log_{10} PFU)			Group 2 (Intramuscular)(Dose = 6.0 Log_{10} PFU)				
	**Animal codes►**	**RH**	**RT**	**GH**	**RH**	**RT**	**GH (Co-housed)**	**GT(Co-housed)**	**W**
	Hyaline cartilage	Unremarkable	Unremarkable	Unremarkable	Unremarkable	Unremarkable	Unremarkable	Unremarkable	Unremarkable
	Adventitia	Unremarkable	Unremarkable	Unremarkable	Unremarkable	Unremarkable	Unremarkable	Unremarkable	Unremarkable
**03.**	**Brain:**								
	Size (cm)	1.0 X 0.8 X 0.7	1.0 X 0.9 X 0.7	1.2 x 1.0 X 0.8	1.0 X 0.8 X 0.7	0.8 X 0.6 X 0.5	0.7 X 0.6 X 0.5	0.8 X 0.6 X 0.5	0.9 X 0.7 X 0.6
	Architecture	Unremarkable	Unremarkable	Unremarkable	Unremarkable	Unremarkable	Unremarkable	Unremarkable	Unremarkable
	Cortex/Greymatter	Unremarkable	Unremarkable	Unremarkable	Unremarkable	Unremarkable	Unremarkable	Unremarkable	Unremarkable
	Subcortical white matter	Unremarkable	Unremarkable	Unremarkable	Unremarkable	Unremarkable	Unremarkable	Unremarkable	Unremarkable
	Blood vessels	Unremarkable	Unremarkable	Unremarkable	Unremarkable	Unremarkable	Unremarkable	Unremarkable	Unremarkable
	Granulomas	Not seen	Not seen	Not seen	Not seen	Not seen	Not seen	Not seen	Not seen
**04.**	**Heart:**								
	Size (cm)	0.9 X 0.7 X 0.5	1.0 X 0.8 X 0.7	0.9 X 0.8 X 0.6	1.0 X 0.8 X 0.7	1.0 X 1.0 X 0.7	1.0 X 0.8 X 0.6	0.9 X 0.7 X 0.5	1.0 X 0.8 X 0.7
	Epicardium	Unremarkable	Unremarkable	Unremarkable	Unremarkable	Unremarkable	Unremarkable	Unremarkable	Unremarkable
	Myocardium	Sparse diffuse mononuclear cell infiltrate	Sparse diffuse mononuclear cell infiltrate	Sparse diffuse mononuclear cell infiltrate	Sparse diffuse mononuclear cell infiltrate	Sparse diffuse mononuclear cell infiltrate	Sparse diffuse mononuclear cell infiltrate	Sparse diffuse mononuclear cell infiltrate	Sparse diffuse mononuclear cell infiltrate
	Endocardium	Unremarkable	Unremarkable	Unremarkable	Unremarkable	Unremarkable	Unremarkable	Unremarkable	Unremarkable
	Chambers	Unremarkable	Unremarkable	Unremarkable	Unremarkable	Unremarkable	Unremarkable	Unremarkable	Unremarkable
	Vessels	Unremarkable	Unremarkable	Unremarkable	Unremarkable	Unremarkable	Unremarkable	Unremarkable	Unremarkable
05.	**Kidney:**								
	Size (cm)	1.0 X 1.0 X 0.8	1.4 X 1.0 X 0.7	1.5 X 1.2 X 0.7	1.5 X 1.0 X 0.8	1.5 X 1.2 X 0.9	1.5 X 1.0 X 0.8	1.5 X 1.3 X 0.7	1.3 X 1.1 X 0.7
	Capsule	Unremarkable	Unremarkable	Unremarkable	Unremarkable	Unremarkable	Unremarkable	Unremarkable	Unremarkable
	Architecture	Maintained	Maintained	Maintained	Maintained	Maintained	Maintained	Maintained	Maintained
	Glomeruli	No significant pathology	No significant pathology	No significant pathology	No significant pathology	No significant pathology	No significant pathology	No significant pathology	No significant pathology
	Tubules	Unremarkable	Unremarkable	Unremarkable	Unremarkable	Unremarkable	Unremarkable	Unremarkable	Unremarkable
	Vessels	No significant pathology	No significant pathology	No significant pathology	No significant pathology	No significant pathology	No significant pathology	No significant pathology	No significant pathology

Supplementary data S1. Histopathological observations

Sr. no.	Groups ►	Group 1 (Intranasal) (Dose = 6.0 Log_{10} PFU)			Group 2 (Intramuscular)(Dose = 6.0 Log_{10} PFU)				
	**Animal codes►**	**RH**	**RT**	**GH**	**RH**	**RT**	**GH (Co-housed)**	**GT(Co-housed)**	**W**
	Interstitium infiltrate	Sparse mononuclear cell infiltrate	Sparse mononuclear cell infiltrate	Sparse mononuclear cell infiltrate	Sparse mononuclear cell infiltrate	Sparse mononuclear cell infiltrate	Sparse mononuclear cell infiltrate	Sparse mononuclear cell infiltrate	Sparse mononuclear cell infiltrate
06.	**Spleen:**								
	Size (cm)	1.3 X 0.5 X 0.2	1.0 X 0.6 X 0.2	1.0 X 0.5 X 0.2	1.0 X 0.6 X 0.2	0.8 X 0.6 X 0.2	1.0 X 0.5 X 0.2	1.0 X 0.5 X 0.2	1.0 X 0.6 X 0.2
	Capsule	Intact	Intact	Intact	Intact	Intact	Intact	Intact	Intact
	Architecture	Maintained	Maintained	Maintained	Maintained	Maintained	Maintained	Maintained	Maintained
	Red pulp	Prominent	Prominent	Prominent	Prominent	Prominent	Prominent	Prominent	Prominent
	White pulp	Lymphoid follicles with secondary germinal centres	Lymphoid follicles with secondary germinal centres	Lymphoid follicles with secondary germinal centres	Lymphoid follicles with secondary germinal centres	Lymphoid follicles with secondary germinal centres	Lymphoid follicles with secondary germinal centres	Lymphoid follicles with secondary germinal centres	Lymphoid follicles with secondary germinal centres
	Fibrosis tissue	Not seen	Not seen	Not seen	Not seen	Not seen	Not seen	Not seen	Not seen
	Granulomas	Not seen	Not seen	Not seen	Not seen	Not seen	Not seen	Not seen	Not seen
	Necrosis	Not seen	Not seen	Not seen	Not seen	Not seen	Not seen	Not seen	Not seen
	Atypical cells	Not seen	Not seen	Not seen	Not seen	Not seen	Not seen	Not seen	Not seen

**01.**	**Lungs:**								
	Size (cm)	1.0 X 0.8 X 0.6	1.4 X 1.3 X 1.0	1.5 X 1.3 X 1.0	1.4 X 1.2 X 1.0	1.4 X 1.0 X 0.8	1.0 X 0.8 X 0.7	1.3 x 1.0 x 0.8	1.3 x 1.0 x 0.8
	Architecture	Unremarkable	Unremarkable	Unremarkable	Unremarkable	Unremarkable	Unremarkable	Unremarkable	Unremarkable
	Alveoli	Unremarkable	Unremarkable	Unremarkable	Unremarkable	Unremarkable	Unremarkable	Unremarkable	Unremarkable
	Stroma	Moderate diffuse mixed inflammatory cell infiltrate along with	Mild diffuse mixed inflammatory cell infiltrate along with	Moderate diffuse mixed inflammatory cell infiltrate along with	Mild to moderate diffuse mixed inflammatory cell infiltrate	Moderate diffuse mixed inflammatory cell infiltrate along with	Moderate diffuse mixed inflammatory cell infiltrate along with	Moderate diffuse mixed inflammatory cell infiltrate along with	Mild diffuse mixed inflammatory cell infiltrate along with

		macrophages.<br><br>100–120 polymorphs / hpf.	macrophages.<br><br>80–90 polymorphs / hpf.	macrophages.<br><br>>200 polymorphs / hpf.	along with macrophages.<br><br>50–60 polymorphs / hpf.	macrophages.<br><br>100–120 polymorphs / hpf.	macrophages.<br><br>100–120 polymorphs / hpf.	macrophages.<br><br>80–90 polymorphs / hpf.	macrophages.<br><br>80–90 polymorphs / hpf.
	Micro-abscesses	Not seen	Not seen	Not seen	Not seen	Not seen	Not seen	Not seen	Not seen
	Alveolar exudate/cells	Not seen	Not seen	Not seen	Not seen	Not seen	Not seen	Not seen	Not seen
	Stromal infiltrate	Seen	Seen	Seen	Seen	Seen	Seen	Seen	Seen
	Other cells (infiltrate)	No granulomas seen	No granulomas seen	No granulomas seen	No granulomas seen	No granulomas seen	No granulomas seen	No granulomas seen	No granulomas seen
**02.**	**Trachea:**								
	Length (cm)	0.8	0.8	1.0	1.0	1.0	0.7	1.0	1.0
	Mucosa	Sparse mononuclear cell infiltrate	Sparse mononuclear cell infiltrate	Sparse mononuclear cell infiltrate	Sparse mononuclear cell infiltrate	Sparse mononuclear cell infiltrate	Sparse mononuclear cell infiltrate	Sparse mononuclear cell infiltrate	Sparse mononuclear cell infiltrate
	Submucosa	Unremarkable	Unremarkable	Unremarkable	Unremarkable	Unremarkable	Unremarkable	Unremarkable	Unremarkable
	Trachealis muscle	Unremarkable	Unremarkable	Unremarkable	Unremarkable	Unremarkable	Unremarkable	Unremarkable	Unremarkable
	Hyaline cartilage	Unremarkable	Unremarkable	Unremarkable	Unremarkable	Unremarkable	Unremarkable	Unremarkable	Unremarkable
	Adventitia	Unremarkable	Unremarkable	Unremarkable	Unremarkable	Unremarkable	Unremarkable	Unremarkable	Unremarkable
03.	**Brain:**								
	Size (cm)	0.7 X 0.6 X 0.5	1.0 X 0.7 X 0.5	1.0 x 0.6 X 0.5	1.2 X 0.9 X 0.7	1.3 X 1.0 X 0.8	1.2 X 1.0 X 0.8	0.8 X 0.7 X0.6	1.0 X 0.9 X0.7
	Architecture	Unremarkable	Unremarkable	Unremarkable	Unremarkable	Unremarkable	Unremarkable	Unremarkable	Unremarkable
	Cortex/Greymatter	Unremarkable	Unremarkable	Unremarkable	Unremarkable	Unremarkable	Unremarkable	Unremarkable	Unremarkable
	Subcortical white matter	Unremarkable	Unremarkable	Unremarkable	Unremarkable	Unremarkable	Unremarkable	Unremarkable	Unremarkable
	Blood vessels	Unremarkable	Unremarkable	Unremarkable	Unremarkable	Unremarkable	Unremarkable	Unremarkable	Unremarkable
	Granulomas	Not seen	Not seen	Not seen	Not seen	Not seen	Not seen	Not seen	Not seen
04.	**Heart:**								

	Size (cm)	1.0 X 0.8 X 0.6	0.8 X 0.6 X 0.5	0.9 X 0.8 X 0.7	0.7 X 0.6 X 0.5	0.8 X 0.7 X 0.6	1.0 X 0.8 X 0.7	1.0 X 0.8 X 0.7	0.8 X 0.6 X 0.5
	Epicardium	Unremarkable	Unremarkable	Unremarkable	Unremarkable	Unremarkable	Unremarkable	Unremarkable	Unremarkable
	Myocardium	Sparse diffuse mononuclear cell infiltrate	Sparse diffuse mononuclear cell infiltrate	Sparse diffuse mononuclear cell infiltrate	Sparse diffuse mononuclear cell infiltrate	Sparse diffuse mononuclear cell infiltrate	Sparse diffuse mononuclear cell infiltrate	Sparse diffuse mononuclear cell infiltrate	Sparse diffuse mononuclear cell infiltrate
	Endocardium	Unremarkable	Unremarkable	Unremarkable	Unremarkable	Unremarkable	Unremarkable	Unremarkable	Unremarkable
	Chambers	Unremarkable	Unremarkable	Unremarkable	Unremarkable	Unremarkable	Unremarkable	Unremarkable	Unremarkable
	Vessels	Unremarkable	Unremarkable	Unremarkable	Unremarkable	Unremarkable	Unremarkable	Unremarkable	Unremarkable
05.	**Kidney:**								
	Size (cm)	1.5 X 1.0 X 0.8	1.5 X 1.2 X 1.0	1.7 X 1.0 X 1.0	1.5 X 1.0 X 0.8	1.5 X 1.2 X 1.0	1.3 X 1.0 X 0.8	1.5 X 1.0 X 1.0	1.5 X 1.0 X 0.8
	Capsule	Unremarkable	Unremarkable	Unremarkable	Unremarkable	Unremarkable	Unremarkable	Unremarkable	Unremarkable
	Architecture	Maintained	Maintained	Maintained	Maintained	Maintained	Maintained	Maintained	Maintained
	Glomeruli	No significant pathology	No significant pathology	No significant pathology	No significant pathology	No significant pathology	No significant pathology	No significant pathology	No significant pathology
	Tubules	Unremarkable	Unremarkable	Unremarkable	Unremarkable	Unremarkable	Unremarkable	Unremarkable	Unremarkable
	Vessels	No significant pathology	No significant pathology	No significant pathology	No significant pathology	No significant pathology	No significant pathology	No significant pathology	No significant pathology
	Interstitium infiltrate	Sparse mononuclear cell infiltrate	Sparse mononuclear cell infiltrate	Sparse mononuclear cell infiltrate	Sparse mononuclear cell infiltrate	Sparse mononuclear cell infiltrate	Sparse mononuclear cell infiltrate	Sparse mononuclear cell infiltrate	Sparse mononuclear cell infiltrate
06.	**Spleen:**								
	Size (cm)	0.7 X 0.7 X 0.2	1.0 X 0.5 X 0.2	0.8 X 0.7 X 0.6	1.3 X 0.5 X 0.2	1.0 X 0.7 X 0.2	1.3 X 0.5 X 0.2	1.0 X 0.7 X 0.2	1.0 X 0.7 X 0.2
	Capsule	Intact	Intact	Intact	Intact	Intact	Intact	Intact	Intact
	Architecture	Maintained	Maintained	Maintained	Maintained	Maintained	Maintained	Maintained	Maintained
	Red pulp	Prominent	Prominent	Prominent	Prominent	Prominent	Prominent	Prominent	Prominent
	White pulp	Lymphoid follicles with secondary germinal centres	Lymphoid follicles with secondary germinal centres	Lymphoid follicles with secondary germinal centres	Lymphoid follicles with secondary germinal centres	Lymphoid follicles with secondary germinal centres	Lymphoid follicles with secondary germinal centres	Lymphoid follicles with secondary germinal centres	Lymphoid follicles with secondary germinal centres
	Fibrosis tissue	Not seen	Not seen	Not seen	Not seen	Not seen	Not seen	Not seen	Not seen
	Granulomas	Not seen	Not seen	Not seen	Not seen	Not seen	Not seen	Not seen	Not seen

	Necrosis	Not seen	Not seen	Not seen	Not seen	Not seen	Not seen	Not seen	Not seen
	Atypical cells	Not seen	Not seen	Not seen	Not seen	Not seen	Not seen	Not seen	Not seen
