# Supplementary material for: A Live Attenuated COVID-19 Candidate Vaccine for Children: Protection against SARS-CoV-2 Challenge in Hamsters
Source: Vaccines (Basel). 2023 Jan 24;11(2):255. doi: 10.3390/vaccines11020255 (PMC9965573; doi:10.3390/vaccines11020255)
Supplement: Supplementary file 1 [file vaccines-11-00255-s001.zip › S6 RNA copy numbers in nasal swabs of challenged animals.pdf]

Supplementary data S6. S6 RNA copy numbers in nasal swabs of challenged animals

Nasal Swabs were collected from day 91 to day 94 (1-4 days post challenge with wild type CoV – Wuhan strain or Delta strain). RNA was estimated by qRT-PCR

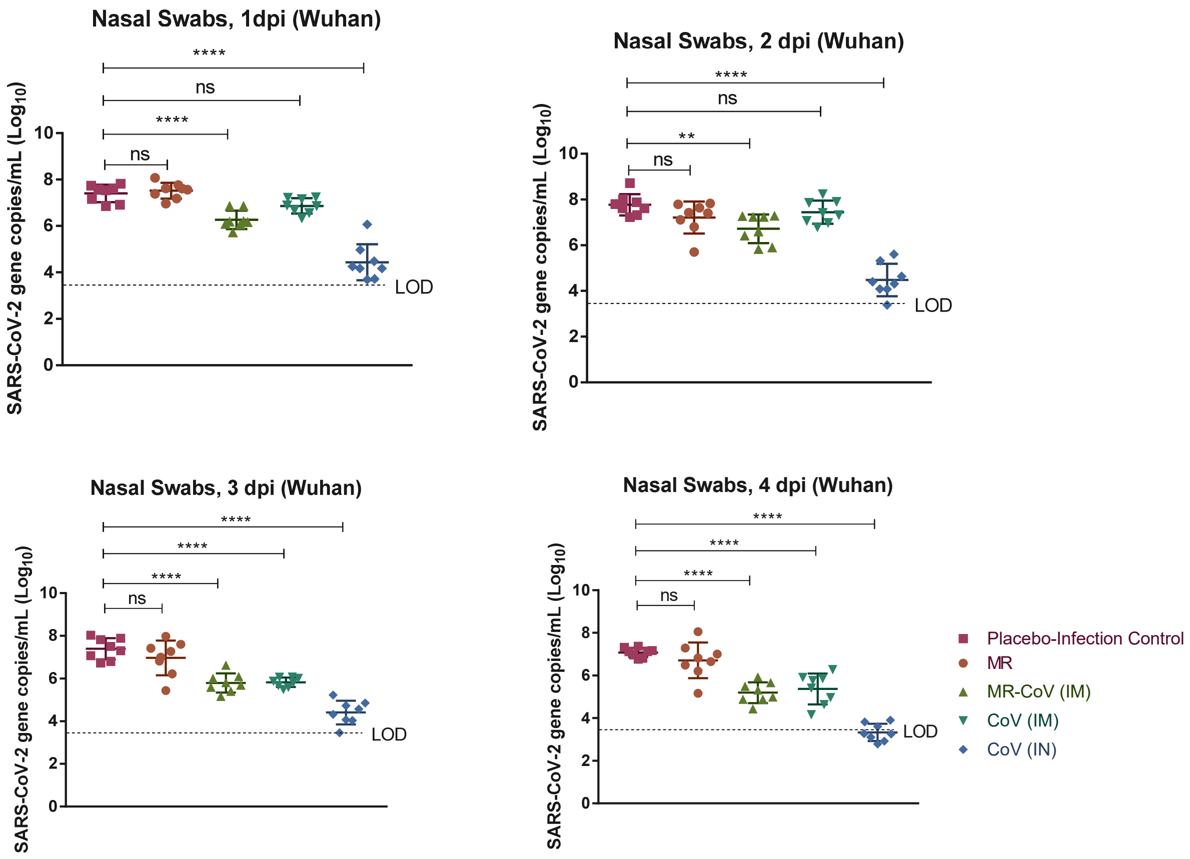

Figure S6a. Viral RNA (Wuhan) in Nasal swabs from day 1 to day 4 post challenge. Error bars = Mean±SD, n=8, One way ANOVA – Dunnets multiple comparison test ** = p≤0.001, **** = p≤0.0001, ns = no significant difference observed.

Supplementary data S6. S6 RNA copy numbers in nasal swabs of challenged animals

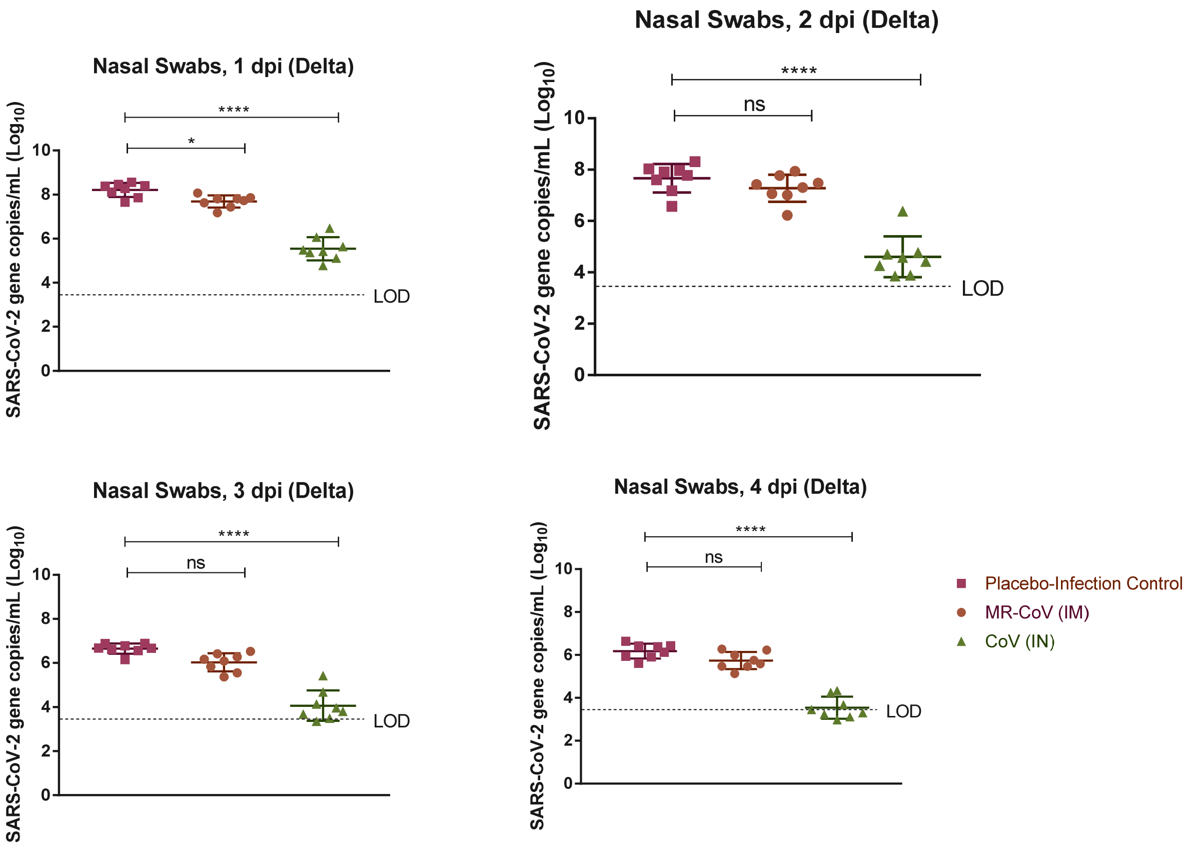

Figure S6b. Viral RNA (Delta) in Nasal swabs from day 1 to day 4 post challenge. Error bars = Mean±SD, n=8, One way ANOVA – Dunnets multiple comparison test * = p≤0.05, **** = p≤0.0001, ns = no significant difference observed.
